# Usefulness of Nonvalved Spacers for Administration of Inhaled Steroids in Young Children with Recurrent Wheezing and Risk Factors for Asthma

**DOI:** 10.1155/2018/3095647

**Published:** 2018-09-03

**Authors:** Carlos Kofman, Alejandro Teper

**Affiliations:** Respiratory Center, Hospital de Niños Dr. Ricardo Gutiérrez, Buenos Aires, Argentina

## Abstract

**Background:**

In vitro and scintigraphic studies have suggested that effectiveness of metered-dose inhalers (MDI) with nonvalved spacers (NVS) is similar to that of MDI with valved holding chambers (VHC). Nevertheless, there are no clinical studies that compare these techniques in long-term treatment with inhaled steroids in young children with recurrent wheezing and risk factors for asthma.

**Objective:**

To compare the efficacy of a long-term treatment with Fluticasone Propionate administered by an MDI through both type of spacers, with and without valves, in young children with recurrent wheezing and risk factors for asthma.

**Patients and Methods:**

Outpatient children (6 to 20 months old) with recurrent wheezing and risk factors for asthma were randomized to receive a 6-month treatment with metered-dose inhaler (MDI) of Fluticasone Propionate 125 mcg BID through an NVS or through a VHC. Parents recorded daily their child's respiratory symptoms and rescue medication use.

**Results:**

46 patients of 13.4 ± 5 months old were studied. During the study period, the NVS group (*n*=25) experienced 3.9 ± 2.4 obstructive exacerbations, and the VHC group (*n*=21) had 2.6 ± 1.6 (*p*=0.031). The NVS group had 17.4 ± 14% of days with respiratory symptoms, and the VHC group had 9.7 ± 7% (*p*=0.019). The NVS group spent 29.8 ± 22 days on albuterol while the VHC group spent 17.9 ± 11 days (*p*=0.022).

**Conclusion:**

Long-term treatment with inhaled steroids administered by MDI and NVS is less effective than such treatment by MDI and VHC in infants with recurrent wheezing and risk factors for asthma.

## 1. Introduction

Asthma is one of the most common chronic diseases in childhood. Inflammation and bronchial hyperreactivity determine recurrent episodes of bronchial obstruction that may be triggered by various stimuli. Antiinflammatory treatment reduces bronchial reactivity and diminishes respiratory events in patients with asthma [1]. In infants with recurrent wheezing and risk factors for asthma, the clinical and functional efficacy of 3-month and 6-month treatments with inhaled steroids has been assessed [2–5].

Inhalation of medication has many advantages in treating diseases of the respiratory tract. Lung deposition of aerosolized particles allows medication to be delivered directly to its site of action, giving a faster onset and allowing smaller doses of drug to be administered. This route determines that systemic absorption of the drug is less and therefore, side effects are usually reduced. Infants and young children cannot coordinate a proper inspiratory maneuver, do not cooperate, and may cry during aerosol administration [6]. For these patients, only two aerosol therapy systems are practical: nebulizers and metered-dose inhalers (MDI) with spacers, both through a facemask [7]. Nebulizers are more time-consuming and have poorer adherence for long-term treatments. Use of an MDI with the spacer is easy and quick and thus is more suitable for infants and young children. Spacers may be designed as simple tubes or as valved holding chambers (VHC). VHCs are capable of retaining the aerosol inside for a brief time after actuation [8], allowing inhalation of more medication, particularly when inspiration is not synchronized with actuation [9], making them especially useful for treating infants and children.

To reduce treatment costs, it has been proposed that homemade nonvalved spacers (NVSs) can be used, assuming that their effectiveness is similar to that of VHC. In vitro [10] and scintigraphic [11] studies have suggested that even simple plastic bottles produce an aerosol deposition similar or even superior to that obtained from a conventional small volume valved spacer. Nevertheless, no published studies have addressed the clinical efficacy of inhaled steroids administered with NVS. The working hypothesis of this study was that NVSs are less efficient than VHC. The main objective was to compare the clinical response of a long-term treatment with Fluticasone Propionate (FP) administered by MDI with NVS versus VHC in young children with recurrent wheezing and risk factors for asthma.

## 2. Materials and Methods

This experimental, randomized, single-blind trial was performed at the Hospital de Niños Ricardo Gutiérrez in Buenos Aires, Argentina, between April 2012 and February 2014. Patients were assessed during treatment by clinical evaluation. The study was approved by the hospital's education and research committee. The parents signed an informed written consent for their child.

To include only children at risk of having asthma, the inclusion criteria were as follows: outpatients attending the respiratory center of the hospital with an age 6 to 20 months; recurrent wheezing, defined as three or more previous episodes of wheezing with clinical improvement after bronchodilators (assessed by a physician), together with a familial history of asthma or any other clinical finding indicating atopy (e.g., allergic rhinitis or eczema) in one or both parents and or a personal history of allergic dermatitis. Patients were included when they met eligibility criteria, had not suffered an acute exacerbation in the last 3 weeks, and had not received any corticosteroid (inhaled or systemic) over the last month.

Patients were excluded if they had a history of severe respiratory infection, diagnosis or suspicion of another chronic pulmonary illness (cystic fibrosis, bronchopulmonary dysplasia, or bronchiolitis obliterans), gastroesophageal reflux, cardiopathy, bronchopulmonary malformations, or thoracic wall deformations.

Patients were not considered for the analysis if they did not follow the treatment regimen or did not attend the control visits on the established dates, or if their symptoms deteriorated enough to require a change in the therapy scheme.

### 2.1. Treatment

Patients were randomized to one of two groups by means of a concealed, computer-generated randomization system performed by a person not otherwise involved in the study. Patients in one group received the medication by means of a 145 cc plastic spacer with a facial mask and two unidirectional valves (Aerochamber Plus Flow-Vu™, Trudell Medical, Canada). Patients in the other group received the medication through a commercial plastic cylindrical NVS of 200 cc with an incorporated face mask (Aeromed™, P. Cassará, Argentina).

Each patient was given an MDI of Fluticasone Propionate (FP) 125 mcg per puff (Flixotide™) and an MDI of albuterol (Ventolin™). Inhalers were provided by GlaxoSmithKline (Argentina). Parents were instructed to administer two doses of FP daily, one in the morning and one in the evening, for 6 months. They also were advised to administer albuterol as needed when the patient presented respiratory symptoms (persistent cough and/or wheezing) and to come to the clinic for unscheduled visits in case of respiratory exacerbations. Parents were instructed on appropriate use of inhalation therapy. With the child in the upright position, the mask was to be hermetically held on the child's mouth and nostrils for 10 seconds after actuation. The spacer devices were to be washed with warm water and detergent every week. Investigators were blinded to which type of spacer each patient was using. Parents were trained at the initial visit and checked at every visit on the proper use of the MDI and spacer by personnel not involved in the study.

### 2.2. Clinical Assessment

Parents were also trained to record their child's day and night symptoms (cough, wheezing, and awakenings due to a respiratory condition), use of rescue medication (number of puffs of albuterol), and number of nonscheduled emergency care visits by filling in a daily card. Presence of symptoms and usage of rescue medication for 3 or more consecutive days were considered as a respiratory exacerbation. The investigator decided when to use systemic steroids.

Clinical appointments were made every 30 days in order to review the daily card, conduct check-ups, review the medicine administration technique, and change used MDIs for new ones. If substantial mistakes in filling out the daily card were detected, patients were disenrolled from the study. Also, compliance with the treatment regimen was assessed in every visit by gravimetry of aerosol canisters using an electronic scale (Mettler Toledo™ PB602-S, USA). Parents were unaware of this procedure. To continue with the protocol, more than 75% of the prescribed dose had to be used during the previous month.

### 2.3. Statistical Analysis

Percentage of days with respiratory symptoms and number of respiratory exacerbations were considered the main variables of the study. The between-group comparisons for clinical variables were analyzed by applying the following algorithm: first, each variable was tested for normality or log normality distribution by using the Shapiro–Wilk test and for homoscedasticity by using the F test. If normality and homoscedasticity were found for a variable, the Student's *t*-test for the between-group comparison was applied. If not, the Mann–Whitney *U* test was used.

A *p* value less than 0.05 was considered significant. A beta type II error of 0.20 indicated that there was no difference between parameters.

## 3. Results

Fifty-one patients fulfilled the inclusion and exclusion criteria. Data of 5 children were excluded from the analysis for the following reasons: 2 patients, one in each group, exhibited poor compliance with treatment; 2 children from the NVS group required hospital admission for acute bronchial obstruction episodes; and one child of the VHC group was removed from the study by parental decision. Demographic characteristics of the 46 children included in the analysis are shown in Table 1.

**Figure 1:**
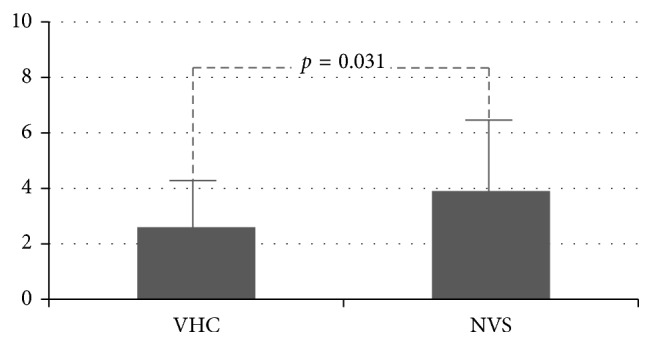
Number of obstructive episodes of bronchial obstruction for children receiving FP 125 mcg BID with MDI plus VHC or NVS for 6 months.

**Figure 2:**
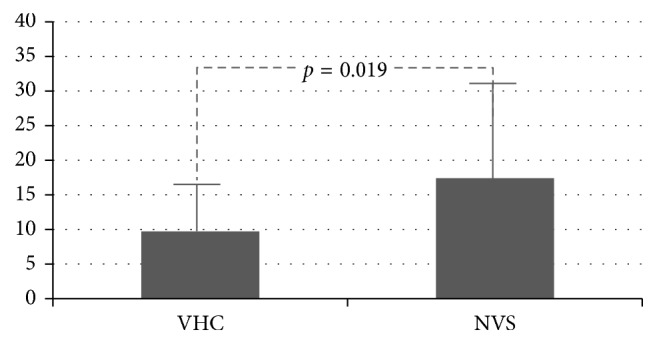
Percentage days with respiratory symptoms of children receiving FP 125 mcg BID with MDI plus VHC and NVS for 6 months.

**Figure 3:**
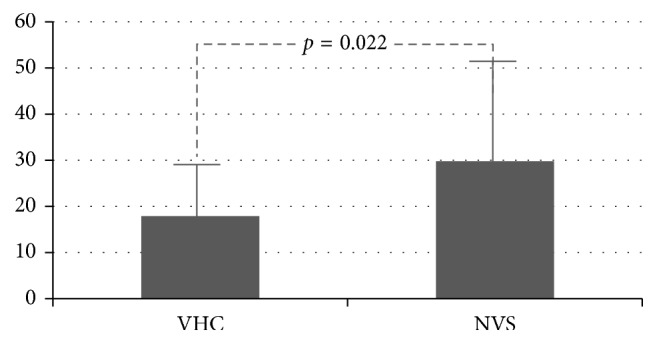
Days on albuterol of children receiving FP 125 mcg BID with MDI plus VHC and NVS for 6 months.

Mean number of obstructive exacerbations was 3.9 ± 2.4 for the NVS group and 2.6 ± 1.6 for the VHC group (*p*=0.031) (Figure 1). Percentage of days with respiratory symptoms was 17.4 ± 14 for the NVS group and 9.7 ± 7 for the VHC group (*p*=0.019) (Figure 2). Number of days on albuterol was 29.8 ± 22 for the NVS group and 17.9 ± 11 for the VHC group (*p*=0.022) (Figure 3). Mean number of courses of systemic corticosteroids was 1.0 ± 1.2 for the NVS group and 0.8 ± 0.9 for VHC group (*p*=0.25).

## 4. Discussion

The present study was conducted to evaluate, through clinical parameters, the usefulness of NVS for long-term inhaled corticosteroid treatment in infants with recurrent wheezing and risk factors to develop asthma. Results demonstrate that clinical response of FP inhaled by an MDI with an NVS is significantly worse than administration of the same drug through a VHC in these patients.

When MDIs are used directly without any spacer, there is a substantial oropharyngeal deposition of the aerosol and significant systemic effects after gastrointestinal absorption may occur [12]. In addition, it is not possible to use MDIs properly in uncooperative patients, such as infants which determine that they can only be treated with spacers. Even using a spacer, young children breathe at tidal volume with no ability to hold the breath at the end of each inspiration which decreases sedimentation of inhaled particles in the distal airways. Different models of spacers available in the market differ in number of valves, design, size, material, and price, and the difference in performance between them can be very high [13–15]. Several studies have stated the efficacy of VHCs not only in generating aerosols with a higher proportion of particles in the respirable range (diameter less than 4.7 microns) than using an MDI alone but also in reducing oropharyngeal deposition, and in maintaining a good delivery of particles even if actuation of the MDI is not synchronized with inspiration [16–19]. With double-valved VHCs, the inspiratory valve closes during exhalation, preventing the entry of the exhaled air into the chamber and diminishing aerosol loss through the back of the spacer. Particles remain suspended for a short time, ready to be inhaled with the next inhalation [20]. By contrast, NVS do not preclude entry to the mouth of particles that are too large and moving too fast to be impacted to the oropharynx. The lesser efficacy observed with the NVS used in this study could be explained by (a) the expiratory flow into the spacer, which drags outside the remaining particles through the spaces between the canister and the actuator or between the actuator and the back hole of the spacer; (b) an incomplete sealing of the mask on the infant's face; and (c) a high variability of the lung dose determined by a lack of coordination between actuation and inspiration. When an NVS is used, reduction in the pulmonary dose is significant (more than two-thirds) if the patient delays inhalation only 1 second with respect to the actuation [8]. Moreover, if actuation occurs during exhalation, pulmonary deposition is up to 90% less than that could be achieved with a synchronic maneuver [12]. By contrast, using a VHC, the influence of timing of actuation is less significant, especially when plastic devices are previously washed with detergents or when they are made with electrostatic dissipative materials [17].

Although every spacer reduces oropharyngeal deposition, this reduction in some cases results from the spacer producing a smaller emitted dose. In vitro studies performed with an impactor cascade give an idea of the dispersion of aerosol particle size and allows establishing a theoretical relationship between pulmonary/oropharyngeal deposition. With these types of studies, the efficiency of the systems in reducing oropharyngeal deposition and in maintaining or even increasing the pulmonary dose can be estimated. For example, Wilkes et al. [13] demonstrated by this method that MDI+VHCs were up to 10 times more efficient than using the MDI alone.

In patients with low income, it is difficult to purchase commercial VHCs because of their relatively high cost. For that reason, the use of homemade spacers built with plastic bottles or the like is common. The dispersion of particle size generated by NVS has not been extensively evaluated in vitro. Wilkes et al. also pointed out that any spacer, valved or not, delivers particles smaller than those delivered by an MDI alone [13]. Nevertheless, the respirable mass, the pulmonary/oropharyngeal deposition relationship, and the efficiency, even when no coordination between actuation and inspiration is present, considerably vary between the different spacers taken into account. For example, the pulmonary/oropharyngeal deposition relationship of the MDI alone, generally less than 4, increases to approximately 18 with a simple cardboard tube and can reach values up to 35 with a VHC. In another in vitro study, by contrast, plastic bottles of different sizes showed a dispersion of particle size similar to a commercial VHC [9].

Another way to evaluate an inhaler system performance is through pulmonary scintigraphic studies with radioactive particles. Using this method, Zar et al. showed that spacers made with 500 cc plastic bottles allow a pulmonary deposition similar to the considered commercial VHCs [11]. Another approach consists in measuring plasmatic concentration of certain drugs after inhalatory administration. Albuterol bioavailability is in direct relationship with penetration of the medication in distal airways and can be used to compare efficiency between different systems. Fowler et al. observed that maximal and mean concentrations of albuterol achieved with VHCs are slightly higher than those attained with NVS [21].

However, most of the in vitro experiences, and those performed in healthy subjects that do not mirror exactly the clinical conditions of a patient, must be confirmed by clinical or functional trials [22]. The majority of these “in vivo” studies comparing VHC and NVS were performed with bronchodilators [23–26]. Many of them found that both systems are similar, and the cheapest one could be chosen. In our opinion, these results are not conclusive because of one or more of the following methodological reasons: low number of subjects included, nonsensitive endpoints considered, and the use of high doses of beta agonists. In fact, if the comparison is done on the plateau of the dose-response curve, two systems of different efficiency could look like equally effective. By contrast, in a previous study, using a low dose of albuterol (100 mcg), we observed that VHCs produce a significantly higher bronchodilator response than NVS in asthmatic children 6 to 17 years old [27].

To our knowledge, no previously published studies addressed the clinical efficacy of long-term treatments of inhaled steroids with NVS. The present investigation clearly shows that the clinical evolution of patients treated with FP 250 mcg/day by an MDI through an NVS is less favorable than when the same medication is administered by a VHC. Patients using an NVS rather than a VHC exhibited a higher incidence of obstructive episodes and more frequent use of rescue bronchodilators. In addition, in these patients, more systemic steroids were needed and more severe events of bronchial obstruction that required hospital admission were seen, although they did not reach statistical significance, probably because of the sample size.

The present study allows us to conclude that, in infants with recurrent wheezing and risk factors to develop asthma, administration of inhaled corticosteroids with MDI and a NVS results in a worse symptom control compared with administration using a VHC. It is possible to speculate that patients requiring long-term use of steroids using cheaper and less-efficient spacers would need higher doses of medication to achieve similar therapeutic responses, paradoxically increasing the final cost of treatment.

## Figures and Tables

**Table 1:** Demographic characteristics of patients (no statistical difference was found for any parameter between groups).

	NVS group (*N*=25)	VHC group (*N*=21)
Age (months)	13.2 ± 6	12.9 ± 3.7
Gender (m/f)	14/11	17/4
Weight (kg)	10.1 ± 1.8	10.1 ± 1.3
Height (cm)	76.2 ± 7	75.4 ± 4.9
Age at 1st episode (months)	4 ± 2	3.8 ± 2
Number of previous episodes	5.6 ± 3	5 ± 2
Day care attendance (%)	32	29

## Data Availability

The source data that were used for this study are available for revision.
